# 

**Published:** 1879-03

**Authors:** 


### Books and Pamphlets Received.

Lectures on Clinical Medicine, delivered at the Hôtel-Dieu, Paris. By A. Trousseau. Vol. III. Translated from edition of 1868, By J. R. Cormache, M. D.

A Practical Manual of the Diseases of Children, with a Formulary. By E. Ellis, M. D. Third Edition. Cloth, pp. 213. Wm. Wood & Co., New York. 1879.

A New Treatise on Assimilation and Digestion. By John Wesley Evans. Cloth, pp. 112. Quincy, Ill. 1878.

Diphtheria—Its Nature, Causes, Prevention and Treatment. By J. H. Kellog, M. D. Cloth, pp. 64. Good Health Publishing Co.

Sixth Annual Report of the Secretary of the State Board of Health of the State of Michigan for the fiscal year ending Sept. 30, 1878. Cloth, pp. 355.

Elements of Comparative Anatomy, by Carl Gegenbaur. Translated by F. Jeffrey Bell, B. A. The Translation revised by E. Ray Lankester, M. A. Cloth, pp. 645. London : Macmillan & Co. 1878.

An Introduction to Pathology and Morbid Anatomy. By T. H. Green, M. D. London. Third American Edition, from fourth revised and enlarged English edition. 132 Illustrations. Cloth, pp. 323. Philadelphia: Lindsay & Blakiston. 1878.

Habitual Drunkenness and Insane Drunkards. By J. B. Bucknill M. D. London. Cloth, pp. 103. London: Macmillan & Co. 1879.

Transactions of the American Otological Society, Eleventh Annual Meeting, Newport, R. I., July 24, 1878. Boston:. Houghton, Osgood & Co.

Transactions of the American Ophthalmological Society.

Transactions of the Wisconsin State Medical Society. 1878.

Second Annual Report of the State Board of Health of Colorado. 1877.

On Loss of Weight, Blood-Spitting and Lung Diseases. By Horace Dobell M. D. Cloth, pp. 274. Philadelphia: Lindsay & Blakiston. 1878.

On the Surgery of the Face. By Francis Mason, F. R. C. S. One hundred illustrations. Cloth, pp. 170. 1878. Philadelphia: Lindsay & Blakiston.

Atlas of Diseases of the Membrana Tympani. By H. MacNaughton Jones. Cloth, pp. 28, beside plates. Philadelphia: Lindsay & Blakiston. 1879.

Diphtheria, its Nature and Treatment; Varieties and Local Expression. By Morell Mackenzie, M. D., London. Cloth, pp. 104. Philadelphia: Lindsay & Blakiston. 1879.

Naval Hygiene, Human Health and the Means of Preventing Disease, with illustrative incidents principally derived from naval experience. By Jos. Wilson, M.D., Medical Director of U. S. Navy. Second Edition. Cloth. pp. 274. 1879. Philadelphia: Lindsay & Blakiston.

A Contribution to the Medical Treatment of Chronic Trigeminal Neuralgia By E. C. Seguin, M.D. Reprint from *Medical Record*, Jan. 4, 1879.

Clinical Lecture on Syphilitic Brain Lesions. Reprint from *New York Medical Journal* September, 1878. By E. C. Seguin, M.D.

First Annual Report of the Presbyterian Eye and Ear Charity Hospital, Baltimore. 1878.

Case of Ovariotomy. By Ed. Bork, M.D. (Successful.) Reprint from *St. Louis Medical and Surgical Journal*, April, 1878.

Case of Ovariotomy. By Ed. Bork, M.D. (Unsuccessful.) Reprint from *St. Louis Medical and Surgical Journal.*

Transactions of the Detroit Medical and Library Association, January, 1879.

Report and Resolutions Relating to Sanitary Legislation, Presented to the American Public Health Association at its meeting in Richmond, Va. Nov. 1878.

The Sanitary Protection Association of Newport, R. I.

Placenta Prævia. By G. M. B. Maughs, M.D. Read before the St. Louis Obstetrical and Gynæcological Society, Sept. 19, 1878.

Journal of the Iowa and Illinois Central District Medical Association, Oct. 10, 1878. Rock Island.

Removal of Morbid Growths from the Uterus. By W. S. Ross, Evansville. Ind. Reprint from the *St. Louis Medical and Surgical Journal.*

One Hundred and Sixty-three Cases of Fracture of Neck of Femur, Collected and Reported by H. Wardner, M.D., Cairo, Ill. Reprint from Chicago Medical Journal and Examiner, January, 1879.

Fifty-third Annual Report of Massachusetts Charitable Eye and Ear Infirmary for the year 1878.

On Fracture of the Femur. By Ed. Bork, M.D., with Illustrations.

Eighteenth Annual Report of the Alabama Insane Hospital at Tuscaloosa, October, 1878.

Conclusion of the Board of Experts authorized by Congress to Investigate the Yellow Fever Epidemic of 1878.

New and Original Theories of the Great Physical Forces. By H. R. Rogers, M.D.

Eight Cases of Intra-laryngeal Tumors Removed through the Natural Passages. By J. H. Hartman, M.D., Baltimore.

Aphasia, or Aphasic Insanity, Which? A Medico-legal Inquiry. By D. C. H. Hughes, St. Louis, Mo. Reprint from *American Journal of Insanity* Jan. 1879.

On Gastro-Elytrotomy. By H. J. Garrigues, M.D., with three wood cuts. Reprint from *New York Medical Journal*, November, 1878.

Address of W. O. Daniell, M.D., President of the Medical Association of Georgia, Delivered at Twenty-ninth Annual Meeting. Reprint from Transactions of Medical Association of Georgia, 1878.

Mansil's Almanac of Planetary Meteorology and New System of Sciences, 1879.

### ANNOUNCEMENTS FOR THE MONTH.

### SOCIETY MEETINGS.

Chicago Medical Society—Mondays, March 3 and 17.

West Chicago Medical Society—Mondays, March 10 and 24.

### CLINICS.

MONDAY.

Eye and Ear Infirmary—2 p. m., Ophthalmological, by Prof. Holmes; 3 p. m., Otological, by Prof. Jones.

Mercy Hospital—1:30 p. m., Surgical, by Prof. Andrews.

Rush Medical College—2 p. m., Dermatological and Venereal, by Dr. Hyde; 3 p. m., Medical, by Dr. Bridge.

Woman's Medical College—2 p. m., Dermatological, by Dr. Maynard.

TUESDAY.

Cook County Hospital—2 to 4 p. m., Medical and Surgical Clinics.

Mercy Hospital—1:30 p. m., Medical, by Prof. Hollister.

WEDNESDAY.

Chicago Medical College—1:30 p. m., Eye and Ear, by Prof. Jones.

Eye and Ear Infirmary—2 p. m., Ophthalmological, by Dr. Hotz.

THURSDAY.

Chicago Medical College—1:30 p. m., Medical, by Prof. Quine.

Rush Medical College—3 p. m., Diseases of the Nervous System, by Prof. Lyman; 4 p. m., Diseases of the Chest, by Prof. Ross.

FRIDAY.

Cook County Hospital—2 to 4 p. m., Medical and Surgical Clinics.

Mercy Hospital—1:30 p. m., Medical, by Prof. Davis.

SATURDAY.

Rush Medical College—2 p. m., Surgical, by Prof. Gunn.

Chicago Medical College—2 p. m., Surgical, by Prof. Isham; 3 p. m., Neurological, by Prof. Jewell.

Woman's Medical College—11 a. m., Ophthalmological, by Dr. Montgomery.

Daily Clinics, from 2 to 4 p. m., at the Central Free Dispensary, and at the South Side Dispensary.

